# Siderophore Immunization Restricted Colonization of Adherent-Invasive Escherichia coli and Ameliorated Experimental Colitis

**DOI:** 10.1128/mbio.02184-22

**Published:** 2022-09-12

**Authors:** Romana R. Gerner, Suzana Hossain, Artur Sargun, Kareem Siada, Grant J. Norton, Tengfei Zheng, Wilma Neumann, Sean-Paul Nuccio, Elizabeth M. Nolan, Manuela Raffatellu

**Affiliations:** a Department of Pediatrics, Division of Host-Microbe Systems and Therapeutics, University of California San Diego, La Jolla, California, USA; b Department of Chemistry, Massachusetts Institute of Technology, Cambridge, Massachusetts, USA; c Center for Microbiome Innovation, University of California San Diego, La Jolla, California, USA; d Chiba University-University of California-San Diego Center for Mucosal Immunology, Allergy, and Vaccines (CU-UCSD cMAV), La Jolla, California, USA; New York University School of Medicine

**Keywords:** *Escherichia coli*, gut inflammation, immunization, inflammatory bowel disease, iron utilization, mucosal immunity, siderophores

## Abstract

Inflammatory bowel diseases (IBD) are characterized by chronic inflammation of the gastrointestinal tract and profound alterations to the gut microbiome. Adherent-invasive Escherichia coli (AIEC) is a mucosa-associated pathobiont that colonizes the gut of patients with Crohn’s disease, a form of IBD. Because AIEC exacerbates gut inflammation, strategies to reduce the AIEC bloom during colitis are highly desirable. To thrive in the inflamed gut, *Enterobacteriaceae* acquire the essential metal nutrient iron by producing and releasing siderophores. Here, we implemented an immunization-based strategy to target the siderophores enterobactin and its glucosylated derivative salmochelin to reduce the AIEC bloom in the inflamed gut. Using chemical (dextran sulfate sodium) and genetic (*Il10*^−/−^ mice) IBD mouse models, we showed that immunization with enterobactin conjugated to the mucosal adjuvant cholera toxin subunit B potently elicited mucosal and serum antibodies against these siderophores. Siderophore-immunized mice exhibited lower AIEC gut colonization, diminished AIEC association with the gut mucosa, and reduced colitis severity. Moreover, Peyer’s patches and the colonic lamina propria harbored enterobactin-specific B cells that could be identified by flow cytometry. The beneficial effect of siderophore immunization was primarily B cell-dependent because immunized *mu*MT^−/−^ mice, which lack mature B lymphocytes, were not protected during AIEC infection. Collectively, our study identified siderophores as a potential therapeutic target to reduce AIEC colonization and its association with the gut mucosa, which ultimately may reduce colitis exacerbation. Moreover, this work provides the foundation for developing monoclonal antibodies against siderophores, which could provide a narrow-spectrum strategy to target the AIEC bloom in Crohn’s disease patients.

## INTRODUCTION

Crohn’s disease (CD) belongs to the spectrum of inflammatory bowel diseases (IBD) and is characterized by chronic inflammation of the gastrointestinal (GI) tract. The pathophysiology of CD is complex and involves dysregulated immune responses toward environmental and microbial triggers, disruptions of epithelial barrier integrity, and alterations of the gut microbiota composition in genetically susceptible individuals (1–5). The healthy human gut microbiota is dominated by obligate anaerobic microbes belonging to the phyla *Firmicutes* and *Bacteroidetes*, whereas the oxidative environment of the inflamed gut, such as in IBD, typically results in a dramatically reduced abundance of these organisms (6, 7). By contrast, other microbes, particularly select facultative anaerobic members of the *Enterobacteriaceae* family (order *Enterobacterales*), thrive during inflammation (8). In addition to the typical compositional shifts in IBD-associated microbial communities, specific bacterial species with potentially disease-triggering properties are also enriched in IBD patients (9). Nevertheless, the causal relationships between such microbes and IBD pathophysiology remain a matter of debate (10). Escherichia coli is a prototypical member of the *Enterobacteriaceae* family and encompasses many different strains, including various commensals and human pathogens (11–13). Adherent-invasive E. coli (AIEC) is a functionally distinct and more virulent type of E. coli that is frequently recovered from the inflamed ileal and colonic mucosa of CD patients (14–17). Contrary to traditional pathogenic E. coli, AIEC strains do not harbor a unique genetic signature and, thus, are typically identified by their pathogenic phenotypes. These characteristics include adherence, attachment, and invasion of intestinal epithelial cells, as well as survival and replication within macrophages, all of which are linked to CD progression (18, 19). AIEC is well adapted to the inflammatory environment of the IBD gut, and some of its phenotypic traits have been linked to virulence-associated genes (20). These traits include genes encoding propanediol utilization, adhesion, cell invasion, and iron acquisition, which are overrepresented in AIEC relative to nonpathogenic E. coli strains (15, 21–24).

Even though it is widely accepted that the bloom of AIEC in the inflamed gut is detrimental, there are no treatment options to selectively eradicate AIEC from the gut mucosa of IBD patients, although a few interventions are being tested in ongoing clinical trials (25). Antibiotic treatment in the context of CD remains controversial due to the induction of bacterial dysbiosis and is, thus, restricted to infectious complications or in the postoperative setting (7). In addition, the emerging threat of antimicrobial resistance further complicates the use of antibiotics, and antibiotics have been associated with exacerbation of AIEC infection in IBD patients (26). Vaccines could be major alternatives to antibiotics for AIEC infection. In addition to preventing disease, vaccines generate polyclonal antibody responses and induce cellular immunity, which decrease the risk and emergence of escape mutations (27).

A promising strategy to target *Enterobacteriaceae*, including E. coli, is the development of siderophore-based vaccines (28, 29). Siderophores are iron-chelating secondary metabolites that are biosynthesized under conditions of iron limitation (such as colitis), released by producers to scavenge iron from the environment, and then imported by dedicated uptake machinery to provide iron to the cell. As such, siderophore-mediated iron acquisition is one way that certain bacterial species overcome iron limitation and promote their growth and competition with the gut microbiota (30, 31). Accordingly, siderophores constitute important fitness and virulence factors of many Gram-negative pathogens (31–33). IBD-associated AIEC strains harbor several siderophore and ferric iron uptake genes, which are also present in extraintestinal pathogenic E. coli (15, 19, 34). Enterobactin (Ent) is an archetypal, catecholate-type siderophore broadly utilized by *Enterobacteriaceae*, including E. coli, to acquire iron from the host (35). The biological importance of Ent is exemplified by the coevolution of the host protein lipocalin-2 (Lcn2), which binds to iron-associated Ent (Fe-Ent) and thereby impedes bacterial iron acquisition (36, 37). In addition to Ent, several *Enterobacteriaceae*, including mostly pathogenic E. coli strains, produce C-glucosylated Ent, known as salmochelin (GlcEnt), which is not bound by Lcn2 and thereby enables evasion of this host-mediated antimicrobial response (38).

We have previously demonstrated that immunization of mice with Ent conjugated to the mucosal adjuvant cholera toxin subunit B (CTB) elicits a strong mucosal immune response against microbe-derived Ent and GlcEnt and conferred protection against intestinal infection with Salmonella enterica serovar Typhimurium (29). Because Ent and GlcEnt are also key virulence factors for E. coli (30, 39), and the genes for their synthesis and transport are expressed by AIEC in the inflamed gut (40), we hypothesized that immunization against Ent/GlcEnt may limit the AIEC bloom during colitis. Here, we showed that Ent immunization reduced AIEC colonization of the inflamed gut, particularly the association of AIEC with the intestinal mucosa, and ameliorated colitis in mice. We also identified Ent-specific B cells in the Peyer’s patches and colonic lamina propria of immunized mice, which could be visualized and isolated by a flow cytometry-based approach. These findings set the basis for the development of monoclonal antibodies against siderophores, which could provide a narrow-spectrum strategy to target AIEC in patients with CD.

## RESULTS

### CTB-Ent immunization reduced AIEC colonization and ameliorated colitis in *Lcn2^−/−^* mice.

During infection, Ent is captured by the host protein Lcn2, which limits Ent-mediated iron acquisition and hinders bacterial growth (37). We previously showed that intranasal immunization of mice with Ent conjugated to the mucosal adjuvant cholera toxin subunit B (CTB), hereafter CTB-Ent, elicited specific antibodies against both Ent and GlcEnt (29). Thus, we sought to investigate the role of CTB-Ent immunization in mice that lack a functional Lcn2. The AIEC strain NRG857c (isolated from a CD patient) harbors several genes involved in siderophore production and iron metabolism, including genes for the synthesis and transport of Ent, GlcEnt, yersiniabactin, and aerobactin, and heme uptake (15). To test whether AIEC NRG857c colonized mice in our facility, *Lcn2*^−/−^ mice were administered a single dose of oral streptomycin 24 h before infection with 10^9^ CFU of AIEC NRG857c or mock. Streptomycin depleted the commensal microbiota and thereby facilitated AIEC colonization of the mouse gut (Fig. S1A and B). During AIEC infection, dextran sulfate sodium (DSS) was provided in the drinking water to induce colitis (Fig. S1A to D), thereby driving iron limitation and consequently promoting microbial siderophore secretion. Mice were stably colonized with AIEC throughout the experiments, and AIEC infection in addition to DSS administration further aggravated colonic inflammation than DSS alone (Fig. S1C to D).

10.1128/mbio.02184-22.1 FIG S1 (A) Weight course of *Lcn2*^−/−^ mice during DSS colitis with or without AIEC infection. (B) Fecal AIEC CFU throughout the infection. (C) Colon lengths were determined at the end of the experiments. (D) Representative H&E-stained sections from distal colons of mice along with histological colitis scores. 100× magnification; Scale bars, 200 μm. pi, postinfection. Data represent mean ± SEM (A, C, and D) or geometric mean (B); *, *P* ≤ 0.05. Download FIG S1, TIFF file, 1.9 MB. Copyright © 2022 Gerner et al. 2022 Gerner et al. https://creativecommons.org/licenses/by/4.0/ This content is distributed under the terms of the Creative Commons Attribution 4.0 International license.

**FIG 1:**
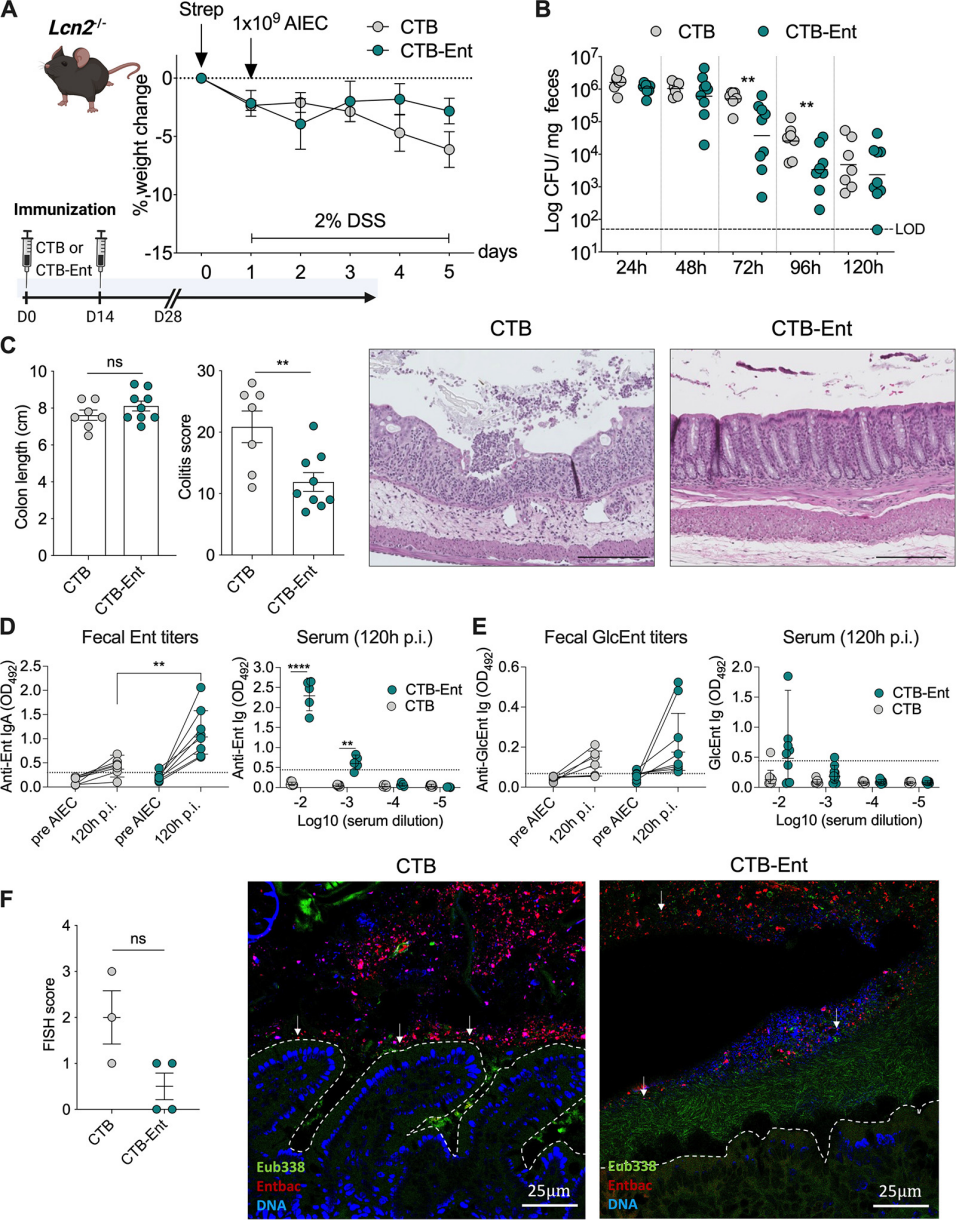
CTB-Ent immunization protected mice from AIEC infection in the absence of Lcn2. (A) Experimental timeline (schematic created with BioRender) and weight loss during AIEC infection in *Lcn2*^−/−^ mice. Mice received 100 μg intranasal CTB or CTB-Ent at day 0, followed by a booster 14 days later. Animals were considered fully immunized 28 days after the initial immunization. (B) Fecal AIEC shedding was monitored in mice immunized with CTB (gray circles) or CTB-Ent (green circles) over time. Each circle represents a mouse. The dashed horizontal line indicates the limit of detection (LOD). (C) Colon lengths and colitis scores were assessed on day 5 post-AIEC infection. Representative pictures from H&E-stained colonic sections are shown. 100× magnification; Scale bars, 200 μm. (D) Optical density 450 nm (OD_450_) values of fecal anti-Ent IgA and blood anti-Ent Ig (120 h pi, dilutions indicated in the figure) are shown. Fecal OD_450_ values are shown from undiluted samples (pre-AIEC) or 1:5 dilutions (120 h pi). (E) OD_450_ values for anti-GlcEnt Ig in feces (undiluted) and serum (120 h pi, dilutions indicated in the figure). Assay cutoffs are indicated by dashed lines. (F) FISH scores represent epithelial attachment and epithelial invasion of AIEC in proximal colon sections from FISH experiments. Representative confocal images of each group are shown. The colonic epithelium is highlighted by a white dashed line. AIEC are indicated with white arrowheads. 200× magnification; Scale bars, 25 μm. LOD, limit of detection; pi, postinfection. Data represent mean ± SEM (A, C, and F), geometric mean (B), or geometric mean ± SD (D and E); ns, not significant; **, *P ≤ *0.01; ****, *P ≤ *0.0001.

Next, we tested whether Ent immunization could mimic the Ent-neutralizing function of Lcn2 in the context of AIEC infection. *Lcn2*^−/−^ mice were immunized with 100 μg CTB-Ent or CTB (mock control) intranasally, followed by a boost after 14 days as described previously (29). After 28 days from the first dose, mice were administered streptomycin 24 h before AIEC infection (14, 16). *Lcn2*^−/−^ mice immunized with either CTB or CTB-Ent during AIEC infection exhibited a similar weight course (Fig. 1A). Analysis of fecal AIEC over time revealed that mice immunized with CTB-Ent exhibited significantly lower AIEC CFU at 72 h and 96 h postinfection (pi) compared to CTB control mice (Fig. 1B). Even though colon lengths were comparable in both groups (colonic shortening is a proxy for intestinal inflammation), mice immunized with CTB-Ent exhibited an overall decreased histological colitis severity (Fig. 1C).

To assess whether CTB-Ent immunization induced specific anti-siderophore antibodies, we evaluated anti-Ent and anti-GlcEnt titers in serum and feces from immunized mice using our in-house ELISA (29), which employed biotinylated Ent or GlcEnt (Fig. S2) bound to streptavidin-coated plates. Both fecal anti-Ent Immunoglobulin (Ig) A and serum anti-Ent Ig were significantly elevated in mice immunized with CTB-Ent after AIEC infection, compared to the CTB control group (Fig. 1D). Fecal and serum anti-GlcEnt titers were also higher in CTB-Ent immunized mice, although this increase was not statistically significant (Fig. 1G).

10.1128/mbio.02184-22.2 FIG S2 Preparation of Biotin-DGE. (A) Structure of Biotin-DGE and analytical HPLC trace of the purified compound. The two glucose moieties are shown in red. Analytical HPLC was performed using a Clipeus reverse-phase C18 column (5 μm, 4.6 × 250 mm; Higgins Analytical, Inc.) and a solvent gradient of 0 to 100% B over 30 min. (B) IroB-catalyzed formation of Biotin-DGE from Biotin-Ent and UDP-glucose. MGE, monoglucosyl enterobactin; DGE, diglucosyl enterobactin (also known as GlcEnt or salmochelin). Download FIG S2, TIFF file, 0.3 MB. Copyright © 2022 Gerner et al. 2022 Gerner et al. https://creativecommons.org/licenses/by/4.0/ This content is distributed under the terms of the Creative Commons Attribution 4.0 International license.

Biofilm formation and association with the intestinal epithelium is a common pathogenic feature of AIEC (41, 42). To visualize the spatial relationship of AIEC with commensal microbes and the gut epithelium, we performed bacterial fluorescence *in situ* hybridization (FISH) from proximal colon samples obtained at 96 h pi. In CTB-immunized mice, AIEC was either located in close proximity to or attached to the intestinal epithelium and within crypts (Fig. 1F). In stark contrast, AIEC was mostly confined to the intestinal lumen of mice immunized with CTB-Ent without adhering to the epithelial layer (Fig. 1F). Instead, microbes detected with the pan-bacterial *Eubacteria* probe (e.g., the microbiota) were adjacent to the epithelial lining and separated AIEC from the mucosa (Fig. 1F).

Together, these data demonstrated that CTB-Ent immunization markedly protected *Lcn2*^−/−^ mice from the development of severe colitis by reducing the colonization and association of AIEC with the gut mucosa. The presence of anti-Ent antibodies also suggested that immunization with CTB-Ent may provide a functional rescue of Lcn2 deficiency.

### CTB-Ent immunization reduced AIEC colonization and colitis in WT mice.

**FIG 2:**
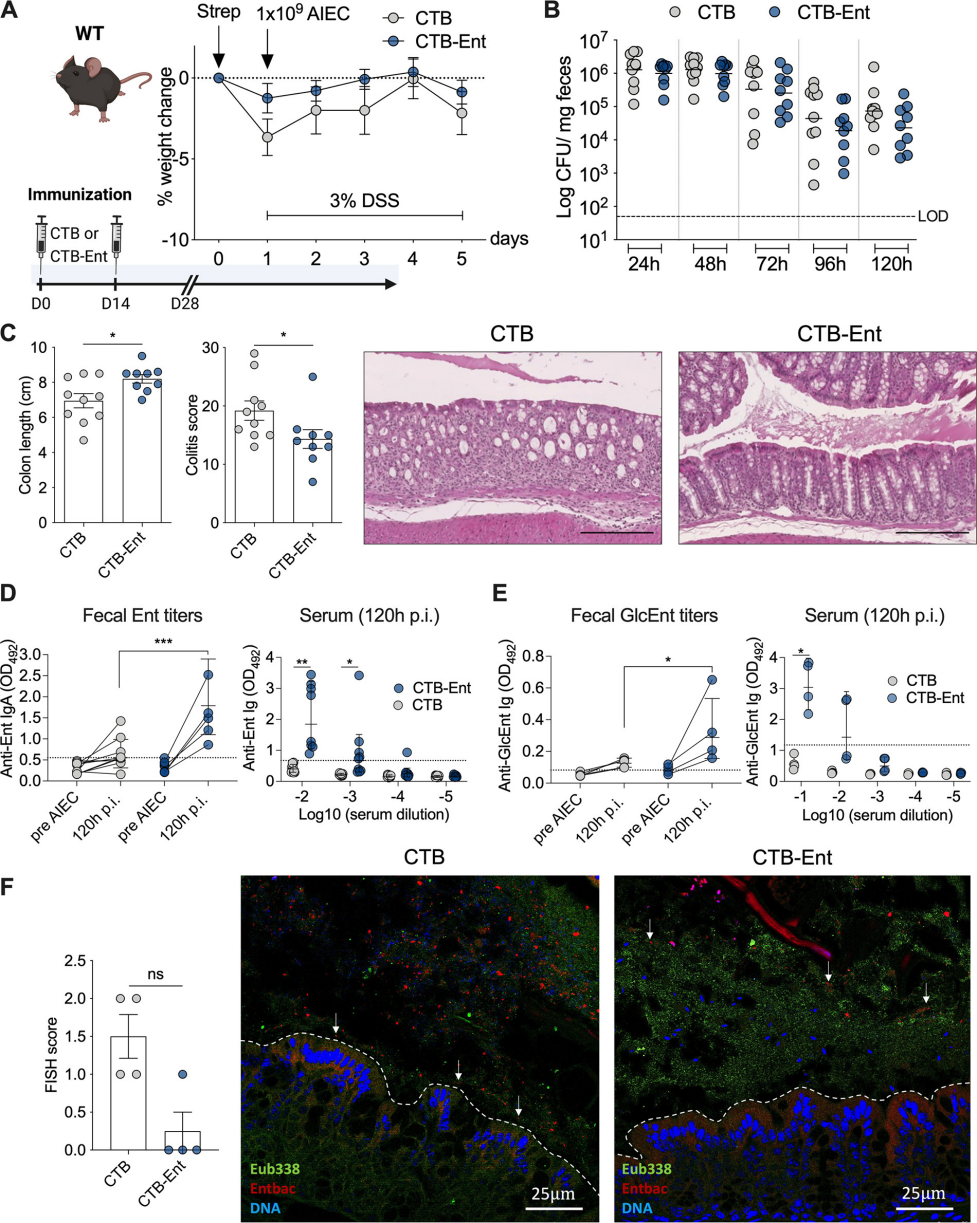
CTB-Ent immunization conferred protection from AIEC-mediated colitis in WT mice (Lcn2 proficient). (A) Experimental timeline (schematic created with BioRender) and weight loss during AIEC infection in WT mice. Mice received 100 μg intranasal CTB or CTB-Ent at day 0 followed by a booster 14 days later. Animals were considered fully immunized 28 days after the initial immunization. (B) Fecal AIEC shedding was monitored in mice immunized with CTB (gray circles) or CTB-Ent (blue circles) over time. Each circle represents a mouse. The dashed horizontal line indicates the limit of detection (LOD). (C) Colon lengths, histopathology colitis scores, and representative H&E-stained colon sections are shown. 100× magnification; Scale bars, 200 μm. (D) Ent-specific IgA levels in feces or Ig in serum are shown as the OD_450_ values. (E) OD_450_ values of fecal and systemic anti-GlcEnt Ig. Fecal anti-Ent OD_450_ values are shown from 1:2 dilutions (at 120 h pi) or undiluted samples (pre-AIEC and GlcEnt). Dashed lines indicate assay cutoffs. (F) Epithelial attachment and epithelial invasion of AIEC in the proximal colon of CTB or CTB-Ent immunized mice were assessed on confocal FISH images and converted into a FISH score. 200× magnification; Scale bars, 25 μm. WT, wild-type; LOD, limit of detection; pi, postinfection. Data represent mean ± SEM (A, C, and F), geometric mean (B), or geometric mean ± SD (D and E); ns, not significant; *, *P* ≤ 0.05; **, *P ≤ *0.01; ***, *P ≤ *0.001.

During IBD and other forms of intestinal inflammation, upregulated Lcn2 production prevents the outgrowth of Ent-dependent microbes (2, 43, 44). Before immunization experiments, we confirmed that wild-type (WT) mice remained colonized with AIEC throughout the experiment (120 h; Fig. S3A and B). Similar to *Lcn2*^−/−^ mice, AIEC infection also exacerbated DSS-induced colitis in WT mice (Fig. S3C). To assess the efficacy of CTB-Ent immunization during AIEC infection in the presence of Lcn2, we immunized and infected WT mice that were littermates of *Lcn2*^−/−^ mice. Following AIEC infection and DSS treatment, both groups exhibited a similar weight course (Fig. 2A) and AIEC colonization levels (Fig. 2B). CTB-Ent immunization, however, resulted in reduced colonic shortening and significantly lower histological colitis scores compared to CTB control mice (Fig. 2C). In line with findings in *Lcn2*^−/−^ mice, CTB-Ent immunization elicited significantly higher levels of anti-Ent (Fig. 2D) and anti-GlcEnt (Fig. 2E) antibodies in feces and serum. FISH analysis from the proximal colon of WT mice immunized with CTB (mock) showed that AIEC was frequently found near the mucus layer or associated with the mucosa (Fig. 2F). In contrast, AIEC was confined to the lumen of WT mice immunized with CTB-Ent (Fig. 2F), akin to what we found in *Lcn2*^−/−^ mice immunized with CTB-Ent (Fig. 1F). Furthermore, AIEC was shielded from the mucosa by a wall of rod-shaped bacteria hybridizing with the *Eubacteria* probe in WT mice immunized with CTB-Ent (Fig. 2F). Thus, Ent immunization protected mice from the development of colitis by limiting AIEC growth and association with the colonic mucosa, even when functional Lcn2 was present.

10.1128/mbio.02184-22.3 FIG S3 (A) Weight course of WT mice during DSS colitis with or without AIEC infection. (B) Fecal AIEC CFU from infected animals. (C) Colon lengths were determined at the end of the experiment and histological colitis scores were obtained from H&E-stained sections from distal colons of mice. 100× magnification; Scale bars, 200 μm. WT, wild-type; pi, postinfection. Data represent mean ± SEM (A and C) or geometric mean (B); *, *P* ≤ 0.05. Download FIG S3, TIFF file, 1.3 MB. Copyright © 2022 Gerner et al. 2022 Gerner et al. https://creativecommons.org/licenses/by/4.0/ This content is distributed under the terms of the Creative Commons Attribution 4.0 International license.

### CTB-Ent immunization protected from aggravated disease in *Il10^−/−^* mice.

To investigate whether Ent immunization is beneficial in a genetic model for IBD, we employed the *Il10*^−/−^ mouse model of colitis (45). Mice with a genetic deficiency for the anti-inflammatory cytokine interleukin (IL)-10 can exhibit a spontaneous bloom of *Enterobacteriaceae* (including E. coli) with the progression of intestinal inflammation; however, this expansion strongly depends on the resident enteric microbiota and the microbial environment of the vivarium (46). *Il10*^−/−^ mice in our animal facility do not develop spontaneous colitis, and AIEC infection by itself only marginally induced colitis (Fig. S4). Microbial siderophore secretion is triggered primarily by inflammation-induced iron restriction. We, therefore, surmised that a beneficial effect of CTB-Ent immunization would especially be observed during more severe intestinal inflammation. Therefore, we utilized a piroxicam-accelerated *Il10*^−/−^ colitis model as previously described (47). Following immunization, mice received a piroxicam-fortified diet for 10 consecutive days before inoculation with 10^9^ AIEC CFU. In this model AIEC colonized mice to high levels without the need for streptomycin pretreatment.

10.1128/mbio.02184-22.4 FIG S4 (A) Weight course of *Il10*^−/−^ mice infected with AIEC or uninfected. (B) Fecal AIEC CFU throughout infection. (C) Histological colitis scores of *Il10*^−/−^ mice at baseline and after AIEC infection along with representative images of H&E-stained sections.100× magnification; Scale bars, 200 μm. UI, uninfected. Data represent mean ± SEM (A and C) or geometric mean (B); *, *P* ≤ 0.05; **, *P ≤ *0.01. Download FIG S4, TIFF file, 1.2 MB. Copyright © 2022 Gerner et al. 2022 Gerner et al. https://creativecommons.org/licenses/by/4.0/ This content is distributed under the terms of the Creative Commons Attribution 4.0 International license.

**FIG 3:**
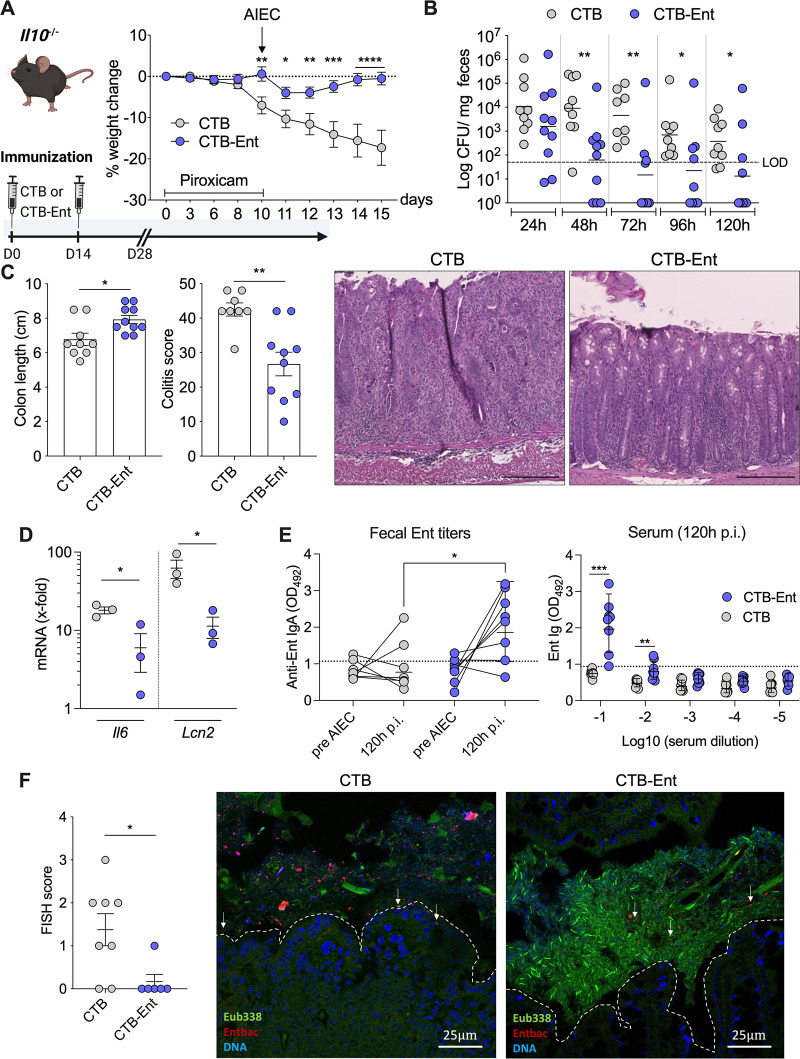
CTB-Ent immunization protected *Il10^−/−^* mice from severe colitis and reduced the AIEC bloom. (A) Experimental timeline (schematic created with BioRender) along with weight loss in piroxicam-treated *Il10^−/−^* mice during AIEC infection. Mice were previously immunized with 100 μg intranasal CTB (gray circles) or CTB-Ent (blue circles) at day 0, followed by a booster 14 days later, and were considered fully immunized after 28 days. (B) AIEC in fecal samples throughout the infection. Each circle represents a mouse. (C) Colon lengths and histological colitis scores were assessed at the end of the experiments. Representative pictures from H&E-stained colonic sections are shown. 100× magnification; Scale bars, 200 μm. (D) Colonic mRNA expression of indicated genes was determined in mice immunized with CTB or CTB-Ent 120 h pi. Data are expressed as fold change in comparison to uninfected mice. (E) OD_450_ values of fecal (undiluted) and systemic (dilutions are indicated in the figure) anti-Ent levels are shown. Assay cutoffs are indicated by dashed lines. (F) FISH scores represent the degree of epithelial attachment and invasion. Representative FISH images of the proximal colon of mice are shown. The epithelium is highlighted by a white dashed line. AIEC are indicated with white arrowheads. 200× magnification; Scale bars, 25 μm. LOD, limit of detection; pi, postinfection. Data represent mean ± SEM (A, C, D, and F), geometric mean (B) or geometric mean ± SD (E); ns, not significant; *, *P ≤ *0.05; **, *P ≤ *0.01; ***, *P ≤ *0.001; ****, *P ≤ *0.0001.

*Il10*^−/−^ mice that were mock-immunized with CTB developed severe disease and lost approximately 17% body weight by day 5 post-AIEC infection (Fig. 3A). In stark contrast, *Il10*^−/−^ mice immunized with CTB-Ent only lost approximately 4% of their body weight 1 day after AIEC infection, and fully recovered by day 5 (Fig. 3A). *Il10*^−/−^ mice immunized with CTB-Ent also showed fewer fecal AIEC beginning at 48 h pi and continuing until the end of experiments. Some immunized mice completely lost AIEC colonization (6 out of 10; Fig. 3B). Colonic shortening as a proxy for intestinal inflammation and histological colitis scores were markedly reduced in *Il10*^−/−^ mice immunized with CTB-Ent compared to CTB controls (Fig. 3C). The differences in colitis corresponded with markedly reduced colonic expression of genes encoding the proinflammatory molecules IL-6 and Lcn2 in the CTB-Ent group compared to CTB control mice (Fig. 3D). Siderophore immunization elicited significant levels of fecal anti-Ent IgA and increased systemic anti-Ent Ig (Fig. 3E). We did not detect GlcEnt Ig titers in these animals (unpublished data).

In agreement with FISH analyses from the DSS colitis model experiments, AIEC was frequently found near or attaching to the intestinal epithelial layer in CTB-immunized control *Il10*^−/−^ mice. In *Il10*^−/−^ mice immunized with CTB-Ent, AIEC was instead primarily located in the colonic lumen (Fig. 3F).

These data indicated that CTB-Ent immunization strongly protected mice from AIEC-induced severe disease and weight loss, reduced AIEC association with the gut mucosa, and ameliorated colonic inflammation in a genetic model for IBD.

### CTB-Ent immunization protected from AIEC infection in a B cell-dependent manner.

**FIG 4:**
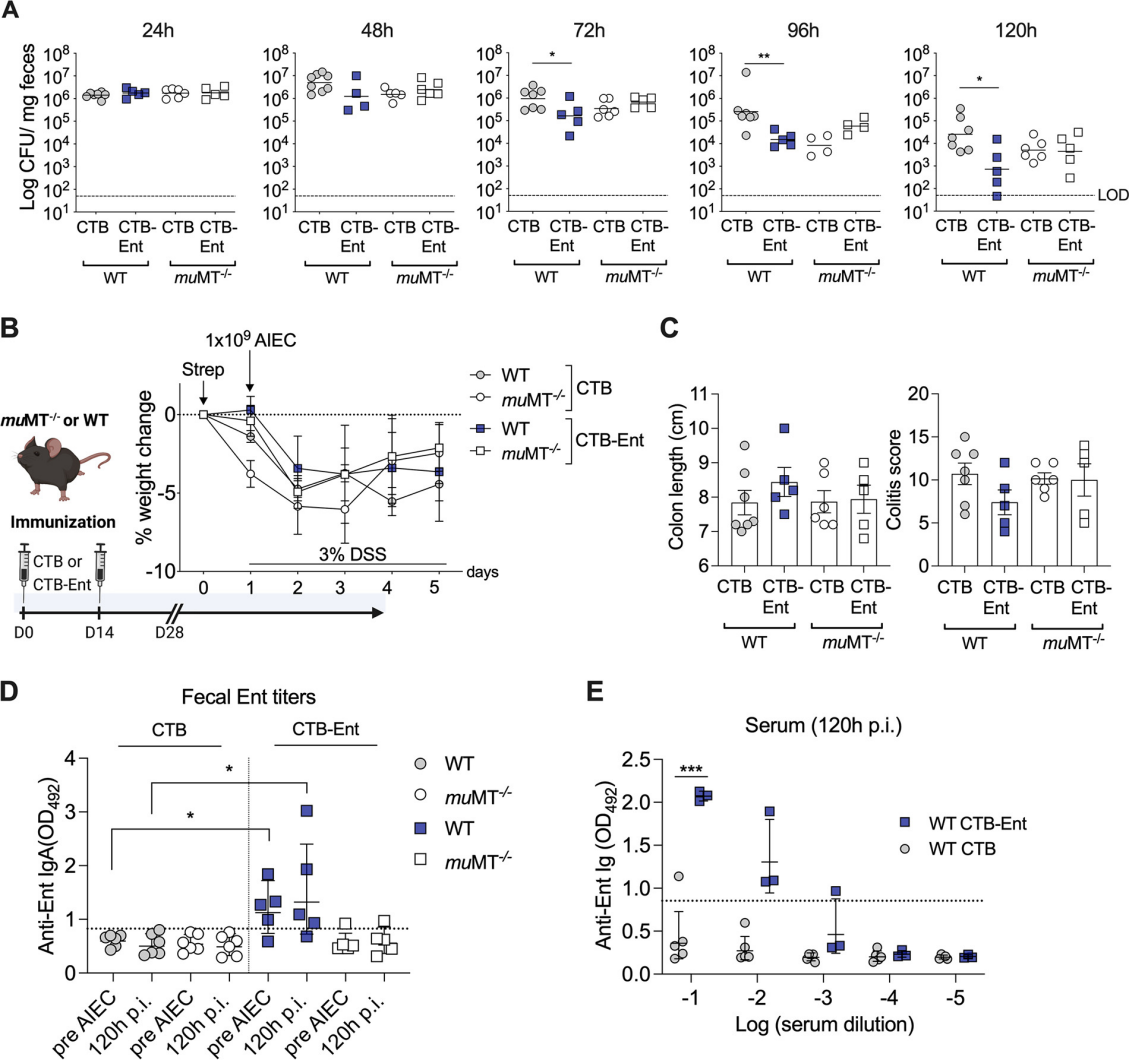
Protection from AIEC infection in mice immunized with CTB-Ent was dependent on B cells. (A) Fecal AIEC of wild-type (WT) and *mu*MT^−/−^ littermate mice that were either immunized with CTB or CTB-Ent before AIEC infection according to the timeline shown in (B). Each symbol represents an individual mouse. (B) Experimental timeline (schematic created with BioRender) along with weight loss during AIEC infection. (C) Colon lengths and histological colitis scores were determined at the end of the experiments. (D) Fecal anti-Ent titers and (E) serum anti-Ent levels of respective groups are shown. Dashed lines indicate the assay cutoffs. WT, wild-type; LOD, limit of detection; pi, postinfection. Data represent geometric mean (A), mean ± SEM (B and C), or geometric mean ± SD (D and E); ns, not significant; *, *P* ≤ 0.05; **, *P ≤ *0.01; ***, *P ≤ *0.001.

As CTB-Ent immunization elicited specific anti-siderophore titers and protected mice from AIEC-induced inflammation, we directly tested whether B cell-conferred humoral immunity was responsible for such a phenotype. We immunized B cell-deficient *mu*MT^−/−^ mice and their WT littermates with CTB or CTB-Ent, followed by DSS administration and AIEC infection. On day 3 to 5 post-AIEC infection, CTB-Ent-immunized WT mice exhibited significantly lower fecal AIEC compared to CTB-immunized WT control animals (Fig. 4A). In contrast, no differences in the levels of AIEC colonization (Fig. 4A) and weight loss (Fig. 4B) were observed between *mu*MT^−/−^ mice immunized with either CTB or CTB-Ent. Although there was a trend of longer colons and lower colitis scores in CTB-Ent-immunized WT animals, this difference did not reach statistical significance (Fig. 4C). WT mice immunized with CTB-Ent displayed specific anti-Ent antibodies in serum and feces (Fig. 4D and E), which were not detected in CTB-immunized WT mice (Fig. 4D and E) or *mu*MT^−/−^ littermates (Fig. 4E, serum data unpublished). Altogether, these data indicated that CTB-Ent immunization triggered B cells to produce anti-Ent antibodies, which resulted in a significant reduction of the fecal AIEC burden in mice.

### Siderophore-specific B cells were identified by a flow cytometry-based approach.

**FIG 5:**
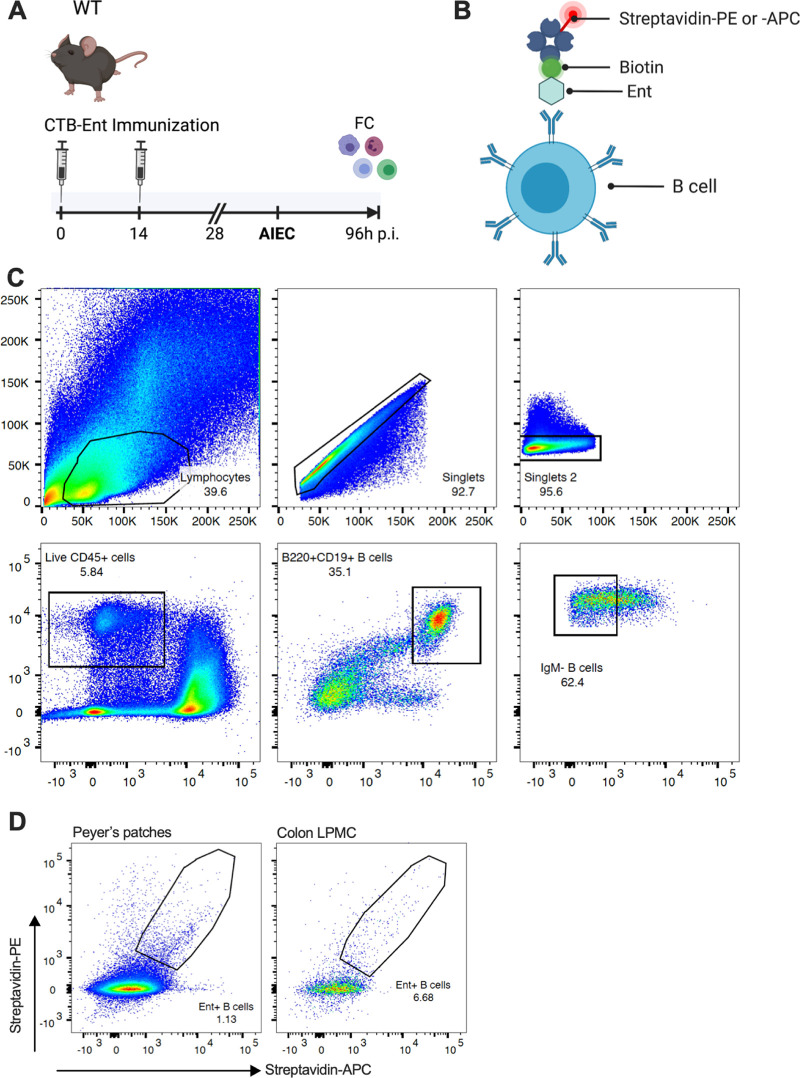
Ent-specific B cells were identified by flow cytometry. (A) WT mice were immunized and infected with AIEC as shown in the schematic (created with BioRender). At 96 h pi, colon lamina propria mononuclear cells (LPMC) and Peyer’s patches (PP) were prepared for flow cytometry (FC) staining. (B) Schematic showing how Ent-specific B cells that recognize biotinylated Ent are detected by incubation with both PE- and APC-conjugated streptavidin to increase sensitivity. Created with BioRender. (C) Flow cytometry gating strategy of cells isolated from PP is shown. After identifying the lymphocyte population and excluding doublets, cells were gated on live CD45^+^ cells followed by identification of CD19^+^/B220^+^ double-positive B cells. After exclusion of IgM^+^ B cells, target B cells (preincubated with Ent-biotin) were identified by PE^+^-Streptavidin/APC^+^-Streptavidin double positivity. (D) Representative plots show Ent-specific B cells in PP and colon LPMC from 4 pooled mice, respectively. LPMC, lamina propria mononuclear cells; PP, Peyer’s patches; FC, flow cytometry; pi, postinfection.

We next aimed to identify and visualize siderophore-specific B cells produced by mice immunized with CTB-Ent. We previously used ELISPOT assays to identify B cells that secreted anti-siderophore antibodies in Peyer’s patches (PP) of mice immunized with CTB-Ent (29). In the gut, PP are key lymphoid structure for the active induction of intestinal immune responses, whereas the lamina propria is the effector site to which activated adaptive immune cells migrate (48, 49). We thus immunized WT mice with CTB-Ent, infected them with AIEC, and collected PP and colons on day 4 pi (Fig. 5A). Single-cell suspensions from PP or colon lamina propria were each pooled from 4 mice and processed for flow cytometry analysis. To identify siderophore-specific B cells, we incubated cells with biotinylated Ent, which we expected to be recognized by the specific surface Ig. Cells were then incubated with two fluorophore-streptavidin conjugates (streptavidin-PE and streptavidin-APC) before staining with surface antibodies (Fig. 5B). Target CD19^+^/B220^+^ B cells were identified from live CD45^+^ cells after exclusion of IgM^+^ B cells (gating strategy outlined in Fig. 5C). In the final gating step, only B cells with a double-positive signal for streptavidin (i.e., both PE and APC) were included to increase specificity. This approach identified Ent-specific B cells in PP (~1.1% of the parent IgM- population) and the colon lamina propria (~6.6% of the parent IgM- population) of immunized mice (Fig. 5D). To our knowledge, this is the first study to identify siderophore-specific B cells in mammals. Moreover, detection of B cells bound to biotinylated siderophores by utilizing fluorescently labeled streptavidin provides a useful approach for fluorescence-activated cell sorting of siderophore-specific B cells in the future.

## DISCUSSION

AIEC is highly prevalent in the gut mucosa of patients with CD, where it is thought to trigger intestinal inflammation (19). Although the first description of AIEC in IBD dates back over 2 decades (50), therapeutic strategies to target AIEC in CD patients are still an unmet medical need. Here, we proposed an immunization strategy to develop antibodies against AIEC siderophores to impede AIEC iron acquisition and thereby hinder AIEC colonization during colitis.

Siderophore immunization elicited mucosal IgA against Ent and GlcEnt. For the first time, we also detected anti-Ent and anti-GlcEnt Ig in the serum, which could play a protective role during systemic infection. Anti-Ent/GlcEnt IgA was produced by B cells localized in small intestinal Peyer’s patches as well as in the colonic lamina propria. The production of anti-siderophore antibodies resulted in decreased fecal AIEC shedding and protected mice from severe intestinal inflammation. Protection was most pronounced in *Il10*^−/−^ mice, which otherwise exhibited profound weight loss and aggravated colitis following piroxicam administration and AIEC infection. The impact of siderophore neutralization might thus be greatest in the context of genetic predisposition and severe intestinal inflammation, which is accompanied by the stringent iron limitation that, in turn, would trigger AIEC to produce more siderophores. Although to a lesser extent than in *Il10*^−/−^ mice, we also observed reduced colitis severity in *Lcn2*^−/−^ and WT mice that were immunized with CTB-Ent, despite similar weight courses, and only a transient decrease of fecal AIEC levels compared to respective control-immunized mice.

The beneficial effect(s) of CTB-Ent immunization on various parameters across experiments could be due to a few reasons, including Ig-mediated blockade of secreted Ent and/or GlcEnt, which, in turn, could explain the reduction of AIEC association with the gut mucosa that we observed by FISH and the consequent reduction of gut inflammation, and Ig-mediated neutralization of the proinflammatory responses induced by secreted Ent and/or GlcEnt.

In the healthy gut, the colonic mucus layer consists of mucins, antimicrobial peptides, and secretory IgA (sIgA), which establish an antimicrobial gradient toward the lumen that deters microbes and toxins from the mucosal surface (51–54). During intestinal inflammation or infection with enteric pathogens, proinflammatory mucosal responses further limit microbial access to the epithelium (55). However, certain pathogens, including AIEC, frequently exploit inflammation and thrive (19, 56). In our CTB-immunized mice, independent of the genotypes (*Lcn2*^−/−^, WT, and *Il10*^−/−^ mice), AIEC was not only found in the gut lumen (reflected by the fecal CFU), but also in close contact with the gut mucosa. In contrast, in mice immunized with CTB-Ent, AIEC was mostly confined to the intestinal lumen and did not come in close contact with the gut mucosa (Fig. 1F, 2F, and 3F). Several studies have demonstrated that the fecal microbiota can differ substantially from mucosa-associated bacterial communities (7, 57–60). In IBD, AIEC and other mucosa-associated microbes have been implicated as important drivers of intestinal inflammation compared to luminal microbes (7). In this context, iron acquisition via siderophores might be particularly critical for sustaining colonization adjacent to the epithelial surface, where the mucus is highly viscous and where iron-restricting antimicrobial proteins, such as Lcn2 and lactoferrin, as well as sIgA concentrations, are highest (51, 53, 54, 61). Thus, Ig-mediated siderophore neutralization may limit the ability of AIEC to closely attach to and/or invade the mucosal surface, resulting in decreased colonic inflammation. Future studies are warranted to further elucidate the exact role of Ent and GlcEnt in such a context.

A second reason that could explain the reduced colitis in mice immunized with CTB-Ent may be due to blockade of proinflammatory responses induced by secreted Ent and/or GlcEnt. Anti-siderophore sIgA likely captures siderophores, rendering them inaccessible to microbes, and neutralizes their proinflammatory properties. This idea is supported by studies undertaken in lung and gut epithelial cells, which demonstrated that iron-free Ent acts as a danger signal, eliciting epithelial IL-8 secretion that is amplified by Lcn2 (62, 63).

Siderophores and their cognate receptors are among the virulence factors employed by AIEC to overcome host barriers and efficiently colonize the gut (15, 19, 40). A recent study utilized an elegant *in vivo* genetic screen and demonstrated that iron acquisition systems were upregulated during AIEC colonization (40). Besides Ent/GlcEnt, other siderophores that evade Lcn2-mediated immunity like aerobactin and yersiniabactin are of critical importance for AIEC virulence (15, 40, 64). For example, yersiniabactin has been shown to promote intestinal fibrosis in *Il10*^−/−^ mice in the absence of binding to its cognate receptor (64).

In our study, anti-siderophore antibodies conferred protection from AIEC colonization and exacerbation of colitis in both *Lcn2*^−/−^ and WT (i.e., Lcn2-proficient) mice. These results might be explained by (i) functional rescue of Lcn2 deficiency by anti-Ent antibodies in *Lcn2*^−/−^ mice, and (ii) antibody-dependent neutralization of GlcEnt (which evades Lcn2) in WT animals. *Il10*^−/−^ mice, however, were strongly protected from AIEC-induced exacerbated colitis even though we only detected anti-Ent antibodies (despite lower titers compared to *Lcn2*^−/−^ and WT mice), but no measurable anti-GlcEnt antibodies. The reduced antibody titers in *Il10*^−/−^ mice might be a consequence of extended administration of piroxicam, a nonsteroidal anti-inflammatory drug (NSAID) that was necessary to induce colitis in our *Il10*^−/−^ colony. NSAIDs have been associated with blunted immune responses, particularly when administered prophylactically (65). Moreover, IL-10 itself is a critical proliferation and differentiation factor for B cells (66), which could also contribute to explaining the decreased antibody titers. Of note, *Il10*^−/−^ mice secrete high levels of mucosal Lcn2 to prevent the outgrowth of Ent-dependent microbes during intestinal inflammation (43). Together, in *Il10*^−/−^ mice, anti-Ent antibodies and Lcn2 could act synergistically to curb AIEC infection and reduce intestinal inflammation. It is also possible that Ent might be essential for certain steps during AIEC infection, despite the presence of Lcn2, and independently of siderophores like yersiniabactin and salmochelin. Thus, the siderophore repertoire of specific AIEC strains might provide context-specific advantages during colitis and dictate the impact of AIEC on host physiology and pathology. Future studies are needed to investigate the relative contribution of other iron uptake systems to AIEC pathogenesis, and whether CTB-Ent immunization conferred greater protection if mice are infected with AIEC strains engineered to only uptake Ent/GlcEnt, but not other siderophores.

Siderophore utilization is not restricted to microbial pathogens. A variety of gut microbes, including nonpathogenic commensals, such as E. coli and Bacteroides thetaiotaomicron, as well as oral *Rothia* spp., employ Ent for iron acquisition (37, 67, 68). We expect that the anti-Ent antibodies generated by our immunization approach are not specific to pathogen-derived siderophores. Thus, it will be essential to investigate the impact of Ent immunization on the gut microbiota composition in the absence of infection or inflammation. In a previous study, we found that immunization of mice with CTB-Ent did not affect the gut microbiota before infection but was associated with an expansion of *Lactobacillus* spp. and a reduction of *Proteobacteria* during Salmonella infection (29). Nevertheless, short-term blockade of siderophores with oral administration of neutralizing antibodies would likely have minimal impact on the commensal microbiota, contrary to the extensive damage of antibiotics on gut bacteria (69).

In conclusion, our work established a proof of concept for siderophores as a valuable target to reduce AIEC-exacerbated colitis and sets the basis for future studies to engineering monoclonal antibodies to target these molecules.

## MATERIALS AND METHODS

### Bacterial strains.

The AIEC strain NRG857c (O83:H1) (14) was obtained from Alfredo Torres (UTMB Health) and Brian Coombes (McMaster University, ON, Canada). For animal infections, AIEC was cultured overnight in Miller Luria broth (LB; 10 g/L NaCl) media containing chloramphenicol (Cm; 0.03 mg/mL) at 37°C with shaking. The following day, bacteria from overnight cultures were washed, diluted, and resuspended in PBS, to a concentration of 5 × 10^9^ CFU/mL.

### Siderophore conjugates.

CTB-Ent and Biotin-Ent were synthesized as previously described (29). Multiple batches of CTB-Ent were prepared and utilized throughout these immunization studies. Biotin-DGE (DGE, diglucosylated enterobactin, also known as GlcEnt or salmochelin) was prepared chemoenzymatically using the C-glucosyltransferase IroB (Fig. S2) (38, 70). A 15-mL solution containing 100 mM Ent-Biotin, 600 mM uridine diphosphoglucose (UDP-glucose, Sigma), 5 mM MgCl_2_, and 1 mM IroB was prepared in 75 mM Tris-HCl buffer at pH 8.0 and divided into 15 ~1000 μL aliquots. These reaction aliquots were incubated at room temperature for 3.5 h and then quenched with the addition of 200 μL 3% trifluoroacetic acid (TFA) (aq). The quenched reaction aliquots were combined in a 50-mL polypropylene falcon tube and lyophilized to dryness. The resulting solid was dissolved in 3 mL of 1:1 MeCN/H_2_O and the resulting mixture was centrifuged (13,000 rpm, 10 min). Biotin-DGE was purified from the supernatant by semipreparative HPLC using an Agilent 1200 series high-performance liquid chromatography (HPLC) instrument outfitted with an Agilent Zorbax reverse-phase C18 column (5 μm, 9.4 × 250 mm) at a flow rate of 4 mL/min and a solvent gradient of 10 to 45% B over 7.5 min (solvent A, filtered Milli-Q water with 0.1% TFA; solvent B, HPLC grade MeCN from EMD Millipore with 0.1% TFA; the method began with a 5 min equilibration at 10% B). The purity and identity of Biotin-DGE were determined by analytical HPLC and high-resolution mass spectrometry (Fig. S2A). [M+H]+ m/z cald. 1519.4941, found 1519.4965.

### Animal immunization and infection.

All animal experiments were approved by the Institutional Animal Care and Use Committee at the University of California, San Diego (protocol number S17107). Specific pathogen-free *Lcn2*^−/−^ mice, *mu*MT^−/−^ mice, their respective wild-type littermates, and *Il10*^−/−^ mice were housed and bred at UCSD. Six- to eight-week-old female and male mice were immunized by intranasal administration of 100 μg of either CTB (Sigma) or CTB-Ent (in PBS), followed by a boost with 100 μg of the same compound 14 days after initial immunization as previously described by our group (29). Before and during infection, mice were regularly monitored for endogenous E. coli by plating fecal homogenates on selective MacConkey agar and CFU compared to numbers derived from Cm plates. No Cm-resistant microbes other than AIEC were detected in our animals. One to 4 weeks after complete immunization, *Lcn2*^−/−^ mice, *mu*MT^−/−^ mice, and their respective wild-type littermates, were administered streptomycin (1 mg/g body weight in PBS) by oral gavage followed by infection with 10^9^ CFU AIEC 24 h later. To induce colitis, 2 to 3% DSS (MP Biomedicals) was provided in the drinking water throughout the infection. *Il10*^−/−^ mice received a piroxicam-substituted diet (100 ppm; Teklad custom research diets, Envigo) for 10 days before infection with 10^9^ CFU AIEC. Weight, fecal AIEC shedding, and anti-Ent/GlcEnt mucosal antibody responses were monitored throughout the experiment. On day 5 pi, mice were euthanized by CO_2_ asphyxiation and gut tissues and blood were collected for further analysis.

### AIEC enumeration and preparation of fecal supernatants for ELISA.

Fresh fecal samples were collected before and after immunization, and daily during AIEC infection. Fecal samples were weighed, resuspended in 1 mL of PBS, and homogenized on a vortex shaker. AIEC was enumerated by plating serial dilutions on LB agar plates containing Cm and incubated at 37°C overnight. For antibody titer quantification, fecal homogenates were centrifuged at 10,000 × *g* for 10 min and the supernatants were collected and stored at −20°C until further analyses.

### Histopathological evaluation.

For histological colitis assessment, mid to distal colon samples were fixed in 10% buffered formalin and processed according to standard procedures for paraffin embedding. The 5 μm sections were stained with hematoxylin and eosin (H&E) and slides were scanned on a NanoZoomer slide scanner and visualized using NDP.view2 viewer software (both Hamamatsu). Colonic inflammation was scored in a blinded fashion using a semiquantitative scoring system as described previously (maximum score = 48) (43).

### Bacterial fluorescence *in situ* hybridization.

Proximal colonic tissue samples were harvested and fixed in Carnoy’s fixative for a minimum of 72 h and then processed as previously described (54). Tissue sections (5 μm) were deparaffinized followed by hybridization for 4 h at 46°C in the presence of 20% formamide utilizing the following probes as reported earlier (71): pan-bacterial EUB338 (5′-GCTGCCTCCCGTAGGAGT-3′), non-EUB (5′-ACATCCTACGGGAGGC-3′) and species-specific ENTBAC (5′-CCTTGCGGTTGGCTTCAGAT-3′). Probes were labeled at the 5′ and 3′ ends with fluorescein isothiocyanate (FITC), Cy5, or Cy3 (BioTez, Berlin, Germany). After washing, cell nuclei were stained with Sytox Deep Red Nucleic Acid Stain (Invitrogen) and cover-slipped using ProLong Gold Antifade Reagent (Life Technologies). Samples were viewed and imaged in a blinded fashion on a Leica DMI4000 B inverted confocal microscope. FISH scores were obtained according to a previously reported scoring system with slight modifications (64). Briefly, epithelial attachment and epithelial invasion of microbes hybridizing with the Cy3-ENTBAC probe were assessed in 3 different regions of the proximal colon sample for each mouse. Bacterial quantities were enumerated as described in (64) and converted to a score from 1 to 4 for both parameters (i.e., a maximum score of 8).

### RNA isolation and qPCR.

Cecal and colonic tissue was snap-frozen in liquid nitrogen and stored at −80°C until further processing. Samples were homogenized with mortar and pestle, and total RNA was extracted utilizing a Qiagen RNeasy Minikit according to the manufacturer’s instructions. Reverse transcription was performed with SuperScript IV VILO Master Mix, and gene expression was analyzed with real-time qPCR by utilizing PowerUp SYBR Green Master Mix and a QuantStudio 5 (all Thermo Fisher). Data were analyzed according to the comparative 2−ΔΔCt method, and target gene expression was normalized to *Actb* mRNA (β-actin). Primer sequences were used as follows: *Actb* 5′-GGCTGTATTCCCCTCCATCG-3′ and 5′-CCAGTTGGTAACAATGCCATGT-3′; *Il6* 5′-GAGGATACCACTCCCAACAGACC-3′ and 5′-AAGTGCATCATCGTTGTTCATACA-3′*; Lcn2* 5′-ACATTTGTTCCAAGCTCCAGGGC-3′ and 5′-CATGGCGAACTGGTTGTAGTCCG-3′.

### Antibody titer quantification by ELISA.

Antibody titers in fecal supernatants and sera were determined by our in-house ELISA as described earlier (29) with minor modifications. Briefly, Streptavidin High Binding Capacity Coated 96-Well Plates (Pierce, Thermo Fisher Scientific) were washed and coated with either biotinylated enterobactin (Biotin-Ent, 1 μg/mL) or salmochelin (Biotin-DGE, 1 μg/m) for 2 h at room temperature (RT). The plates were then washed 3 times with wash buffer (Tris-buffered saline, 0.1% bovine serum albumin [BSA], 0.05% Tween 20), followed by incubation with 100 μL of diluted fecal supernatants or serially diluted sera for 1 h at RT. Subsequently, the plates were washed 3 times and incubated with IgA-HRP or Ig-HRP antibody (both from Southern Biotech; 1:1,000 dilution in wash buffer). Next, 100 μL of the antibody dilution was added to each well, and the plates were incubated for 30 min at RT with gentle rocking. After incubation, the plates were washed 3 times followed by the addition of 100 μL substrate solution (SIGMA*FAST* OPD, Sigma) and incubation for 20 min in the dark. The reaction was quenched by adding 50 μL of 2N hydrogen sulfate (H_2_SO_4_) to each well. Absorbance was read at an optical density (OD) at 492 nm using a Biotek Epoch Microplate Spectrophotometer. The cutoffs for enzyme-linked immunosorbent assays (ELISAs) were determined for each assay according to a previously described method (72).

### LPMC and PP isolation.

Lamina propria mononuclear cells were isolated as previously described with minor modifications (43). Briefly, colons were harvested at 96 h pi, flushed with cold PBS (Gibco), and cut longitudinally. Tissue was minced with a scalpel and shaken in calcium/magnesium-free Hank’s balanced salt solution (HBSS)/10% fetal bovine serum (FBS) (Life Technologies) containing 1 mM DTT and 2 mM EDTA (Sigma-Aldrich) for 20 min at RT. Subsequently, fragments were vortexed for 4 min at full speed, supernatants removed, and tissue fragments resuspended in HBSS/FBS, followed by another vortex step for 2 min. After removal of supernatant (containing epithelial cells), tissue fragments were transferred into IMDM medium (Life Technologies) supplemented with 20% FBS, 128 U/mL type IV collagenase, and 10 U/mL DNase II (both Sigma-Aldrich), and then rotated at 37°C for 60 min. After digestion, samples were resuspended thoroughly. Suspensions were consecutively filtered through 100/70/40 μm cell strainers and spun down at 350 × *g* for 10 min.

For PP isolation, structures were visually identified on the antimesenteric side of the small intestine, dissected with curved scissors, and placed into cold PBS/0.5% BSA (flow buffer, FB). To generate single cell suspensions, PP was then ground into the mesh of a 70 μm nylon mesh cell strainer (Thermo Fisher) using the plunger from a 1 mL syringe (BD Biosciences). The cell strainer was flushed with FB and cell suspensions were centrifuged at 350 × *g* for 10 min, followed by resuspension in FB and further processing for flow cytometry staining.

### Flow cytometry.

All steps were carried out at 4°C in the dark. Single-cell suspensions from PP and lamina propria were blocked for nonspecific binding followed by a viability staining with Zombie yellow (both BioLegend). After washing with FB at 350 × *g* for 10 min, cells were incubated with Ent-Bio for 30 min and washed 3 times with FB. Cells were then incubated with the secondary reagents Streptavidin-PE and Streptavidin-APC for 30 min followed by 3 washes with FB. Surface staining was performed using the following monoclonal antibodies: APC/Cy7 anti-mouse CD45 (clone 30F-11), FITC anti-mouse CD19 (clone 6D5), PerCP/Cy5.5 anti-mouse B220 (clone RA3-6B2), and bv421 anti-mouse IgM (clone RMM-1; all from BioLegend) was done for 30 min, followed by 3 washes with FB. Subsequently, antigen-specific B cells were identified by flow cytometry using a BD LSRFortessa (BD Biosciences), based on a PE^hi/^APC^hi^ double-positive signal. Data were analyzed using FlowJo v10 software.

### Statistical analysis.

Statistical analysis was performed with GraphPad Prism v9. CFU data were log-normalized before statistical testing. Normality distribution was tested with the Shapiro-Wilk test. For comparison between 2 normally distributed groups, *P* values were calculated by unpaired Student's *t* test. Mann-Whitney *U* was performed for unpaired data sets that are not normally distributed. One-way analysis of variance (ANOVA) with Tukey’s multiple-comparison test was used when analyzing more than 2 groups.
