# Supplementary figures and images for: Siderophore Immunization Restricted Colonization of Adherent-Invasive Escherichia coli and Ameliorated Experimental Colitis
Source: mBio. 2022 Sep 12;13(5):e02184-22. doi: 10.1128/mbio.02184-22 (PMC9600343; doi:10.1128/mbio.02184-22)

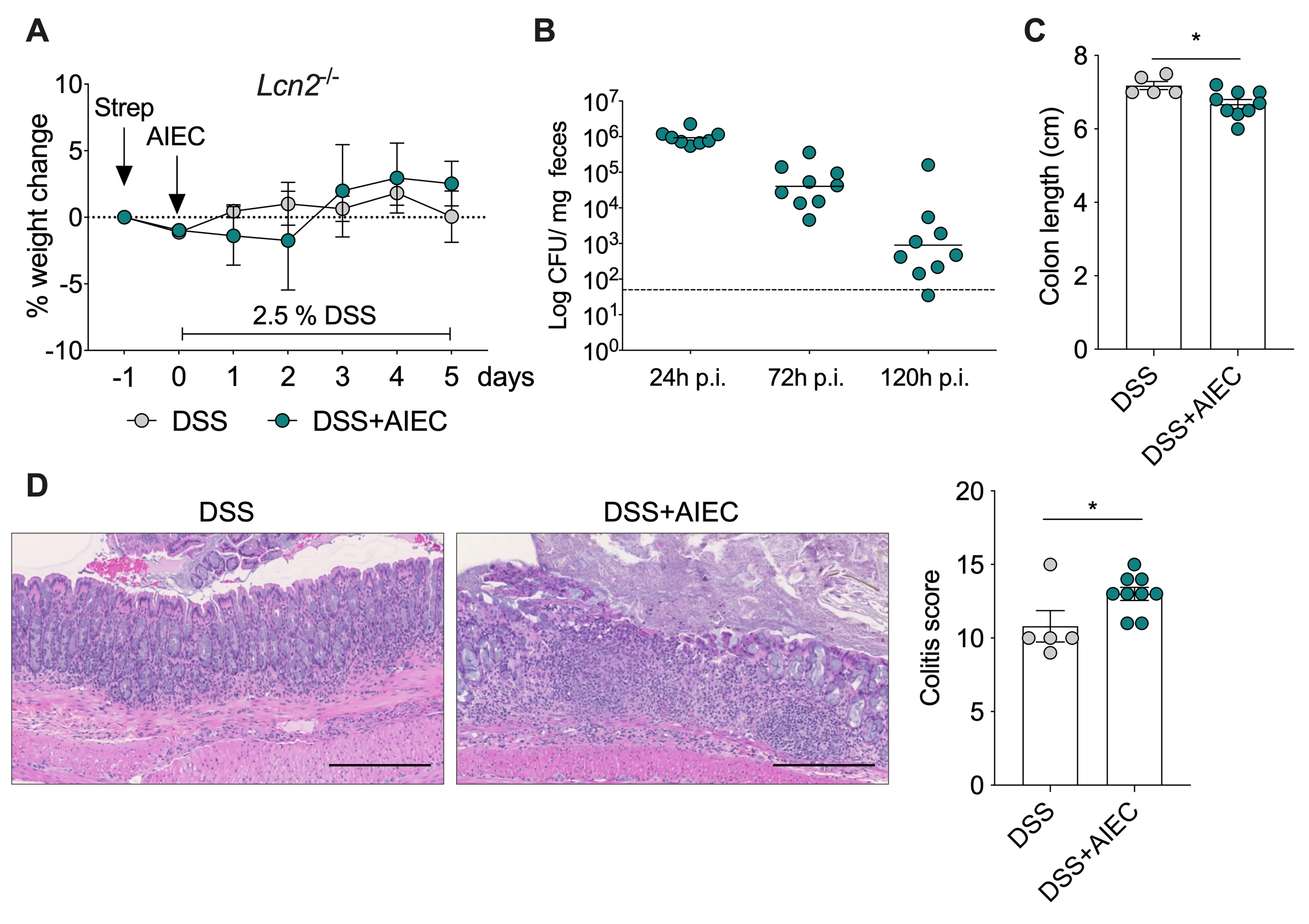

Supplement: FIG S1 [file mbio.02184-22-s0001.tiff]

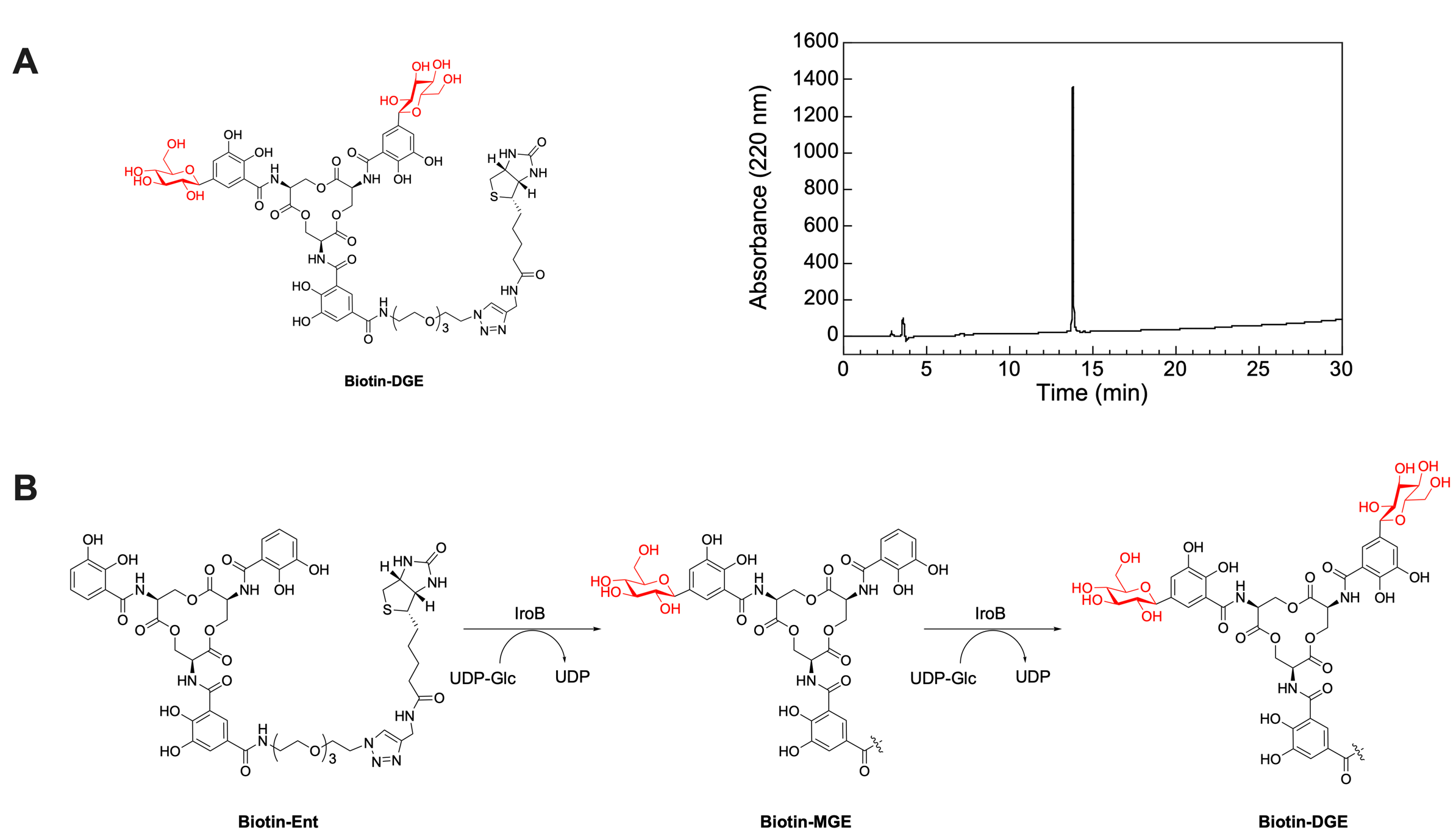

Supplement: FIG S2 [file mbio.02184-22-s0002.tiff]

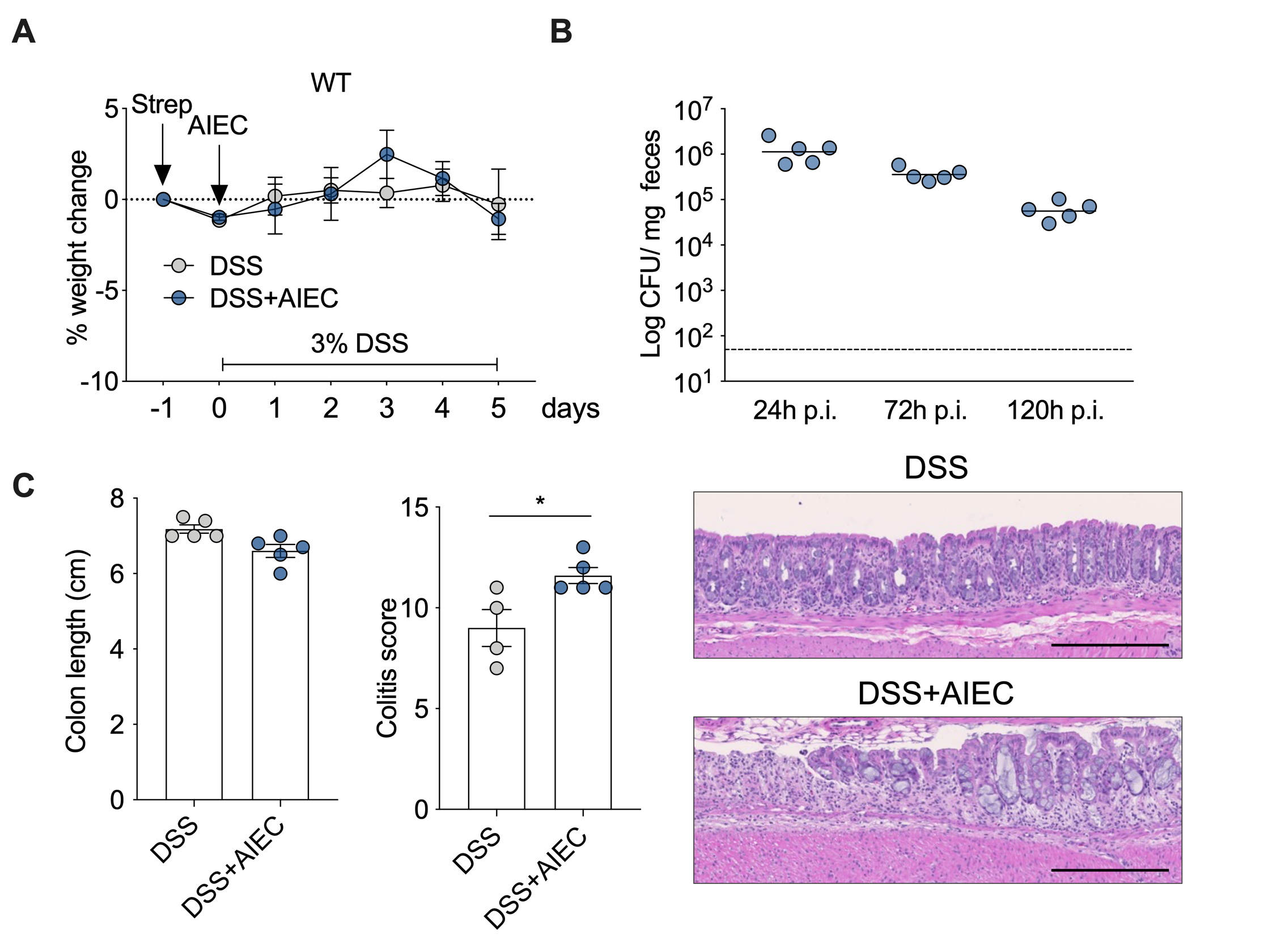

Supplement: FIG S3 [file mbio.02184-22-s0003.tiff]

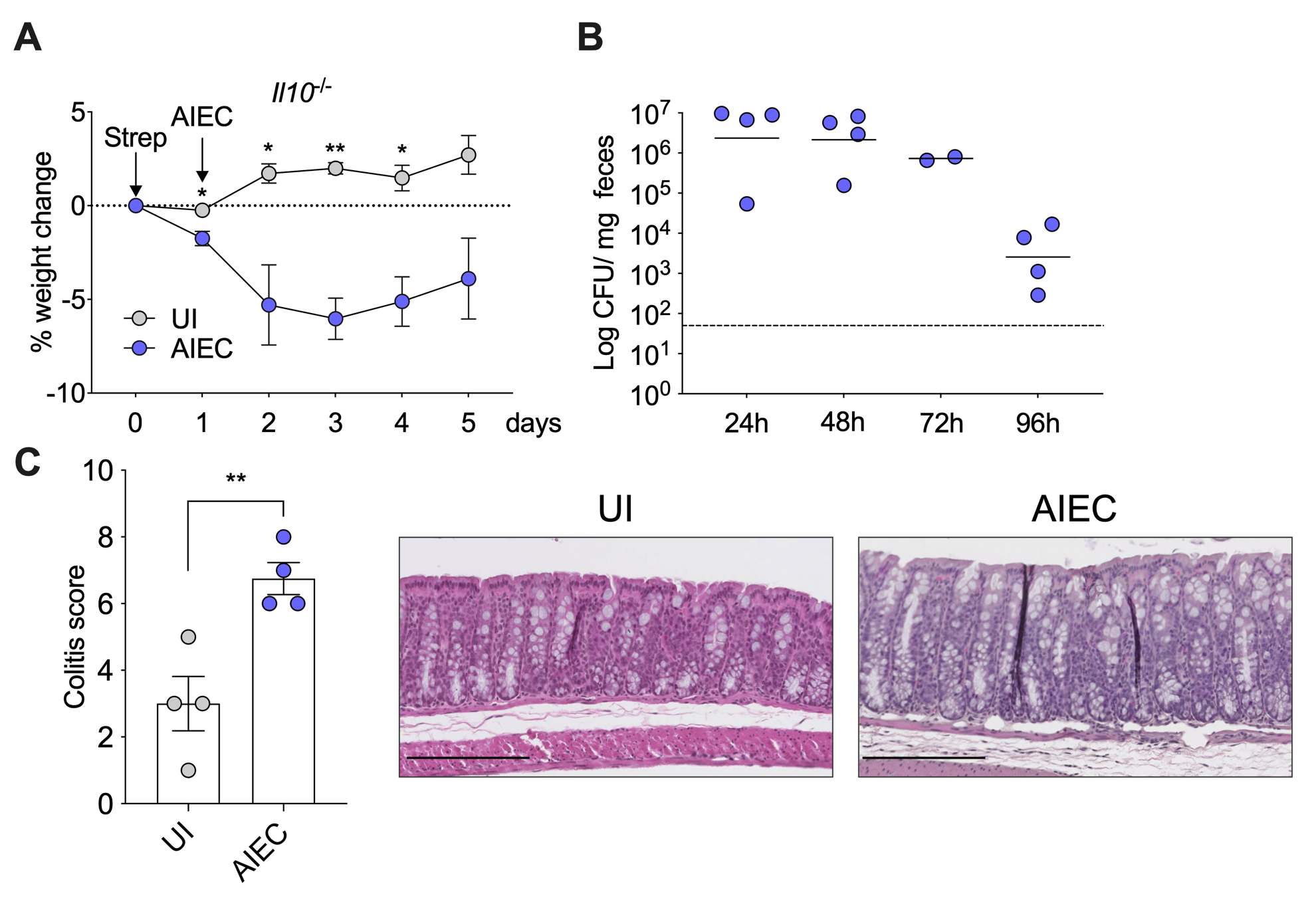

Supplement: FIG S4 [file mbio.02184-22-s0004.tiff]
